# The impact of obstructive sleep apnea on growth in patients with syndromic and complex craniosynostosis: a retrospective study

**DOI:** 10.1007/s00431-022-04621-6

**Published:** 2022-09-28

**Authors:** S. Yang, I. M. J. Mathijssen, K. F. M. Joosten

**Affiliations:** 1 Department of Plastic and Reconstructive Surgery and Hand Surgery, Erasmus Medical Center Sophia Children’s Hospital, Rotterdam, Netherlands; 2 Department of Pediatric Intensive Care, Erasmus Medical Center Sophia Children’s Hospital, Rotterdam, Netherlands

**Keywords:** Growth and development, Growth charts, Craniosynostosis, Obstructive sleep apnea

## Abstract

Given the high prevalence of OSA in children with syndromic and complex craniosynostosis (SCC) and the consequences of untreated OSA, it is important to assess their nutritional status and growth. Yet, literature regarding growth in children with SCC remain scarce. Therefore, this study aimed to (1) illustrate the growth pattern in SCC, (2) determine the impact of OSA on this growth pattern, and (3) evaluate the effect of surgical treatment of OSA on growth over time. A retrospective study was performed in children with SCC, who were treated at the Dutch Craniofacial Center (Rotterdam, Netherlands). Growth variables (height, weight, weight-for-age standard-deviation-score (SDS), weight-for-height SDS, and height-for-age SDS) and degree of OSA (obstructive apnea–hypopnea index) were assessed. Of the 153 children with SCC, 38 (25%) were acutely malnourished at some point during follow-up, of whom 21 had disease-related acute malnutrition. Children with moderate-severe OSA had significant lower weight-for-height SDS compared to children without OSA (*p* = 0.0063). Growth parameters (weight-for-age SDS, weight-for-height SDS, height-for-age SDS) in children with SCC without OSA were not impaired as they did not differ from the normal healthy population, with exception of the patients with Saethre-Chotzen syndrome (SCS) who had a significantly lower SDS for height-for-age.

*   Conclusion*: Children with SCC have a substantial chance of developing acute malnutrition at some point during growth. Additionally, in children with moderate-severe OSA, a significant lower SDS for weight-for-height is present, indicating the importance of assessing the weight and growth pattern in children who are clinically suspected for OSA.

**Table:** 

**What is Known:** *• Obstructive sleep apnea is seen in up to two-thirds of the children with syndromic and complex craniosynostosis.* *• Presence of obstructive sleep apnea is associated with intracranial hypertension and an increased risk of metabolic, cardiovascular, and neurocognitive consequences later in life. Untreated obstructive sleep apnea may lead to impaired growth and weight gain, which can result in growth failure.*	
**What is New:** *• Craniosynostosis patients with moderate-severe obstructive sleep apnea had significant lower weight-for-height standard deviation scores (SDS), compared to children without obstructive sleep apnea. * *• Children with syndromic and complex craniosynostosis without OSA did not significantly differ from the normal healthy population in regard to weight-for-age SDS, weight-for-height SDS, and height-for-age SDS.*

**What is Known:**

*• Obstructive sleep apnea is seen in up to two-thirds of the children with syndromic and complex craniosynostosis.*

*• Presence of obstructive sleep apnea is associated with intracranial hypertension and an increased risk of metabolic, cardiovascular, and neurocognitive consequences later in life. Untreated obstructive sleep apnea may lead to impaired growth and weight gain, which can result in growth failure.*

**What is New:**

*• Craniosynostosis patients with moderate-severe obstructive sleep apnea had significant lower weight-for-height standard deviation scores (SDS), compared to children without obstructive sleep apnea. *

*• Children with syndromic and complex craniosynostosis without OSA did not significantly differ from the normal healthy population in regard to weight-for-age SDS, weight-for-height SDS, and height-for-age SDS.*

## Introduction

Obstructive sleep apnea (OSA) is seen in up to two-thirds of the children with syndromic and complex craniosynostosis (SCC) [1]. It is crucial to diagnose and treat OSA as early as possible as it is associated with intracranial hypertension and an increased risk of metabolic, cardiovascular, and neurocognitive consequences later in life [2–5]. Screening is indicated if clinical symptoms and signs of OSA are present, such as snoring, fatigue, breathing stops during sleep, inattention, and hyperactivity [6]. Gold standard to diagnose OSA in children is the overnight in-hospital polysomnography (PSG), with the home respiratory polygraphy as its alternative [7].

In children with OSA, who are otherwise healthy, OSA is most commonly caused by adenotonsillar hypertrophy. Consequently, the first choice of treatment in these children is the adenotonsillectomy (ATE) [8]. In children with SCC, however, the pathophysiology behind OSA is often multifactorial, as it is associated with midface hypoplasia, adenotonsillar hypertrophy, mandibular hypoplasia, and pharyngeal anomalies [9–11]. Hence, multidisciplinary care is crucial in children with SCC, in which invasive treatment of OSA depends on the level of obstruction and consists of treatment such as midface advancement surgery, mandibular distraction osteotomy, and ATE [9, 12].

Untreated OSA in children is associated with impaired growth and weight gain, which can result in growth failure [13–15]. This association can be explained by increased energy expenditure at night due to increased work of breathing and by the interrupted nocturnal growth hormone secretion of insulin-link growth factor (IGF-1) [16]. Given the high prevalence of OSA in children with SCC and the consequences of untreated OSA, it is important to regularly assess nutritional status and growth in this patient population. Yet, literature regarding growth in patients with SCC remain scarce. Therefore, this study aimed to (1) illustrate the growth pattern in children with SCC, (2) determine the impact of OSA on this growth pattern, and (3) to evaluate the effect of surgical treatment of OSA on growth over time.

## Methods

A retrospective study was performed in children with SCC, who were treated at the Dutch Craniofacial Center (Erasmus University Medical Center Sophia Children’s Hospital, Rotterdam, Netherlands). All clinical care followed a protocolled management, as illustrated in our previous study [12]. The study protocol was exempted from review by the institutional research ethics committee (MEC-2005–273 and MEC-2017–1143). Children were eligible for inclusion if the following criteria were met: (1) children up to 18 years of age with either syndromic or complex (i.e., children with multiple fused sutures, but without confirmation of a genetic mutation) craniosynostosis and (2) at least two measurements of weight and/or height. Children who visited the craniofacial center solely for a second opinion (i.e., who did not receive any medical treatment) were excluded from the study. End of follow-up was defined as last clinical visit, death, or end of study.

### Growth and nutritional status

Nutritional status was determined using height and weight measurements, which were retrospectively gathered using electronic patient files. Height (in cm) and weight (in kg) were measured in a standardized way with calibrated devices and expressed in standard deviation scores (SDS) and evaluated using growth charts, by comparing our data to published standards based on the Dutch pediatric population [17]. Height was measured using a stadiometer (Holtain Limited, Crymych, UK), while weight was measured by an electronic personal scale (SECA, Hamburg, Germany). SDS were calculated for weight-for-height (WFH), weight-for-age (WFA), and height-for-age (HFA) using Growth Analyser RCT (Growth Analyser by Dutch Growth Research Foundation, version 4.1.5). Overall malnutrition was defined if acute malnutrition and/or chronic malnutrition was present (Table 1).

**Table 1:** Definition of nutritional status

**Type of malnutrition**	**Criteria**
**Acute malnutrition** Age < 1 year old Age 1–18 years old Age 0–18 years old	WFA < − 2 SDS WFH < − 2 SDS WFA/WFH deflection of > 1 SDS
**Chronic malnutrition** Age 0–18 years old Age < 4 years old Age > 4 years old	HFA < -2 SDS HFA deflection of > 0.5 SDS within 1 year HFA deflection of > 0.25 SDS within 1 year

*WFA* weight-for-age, *WFH* weight-for-height, *HFA* height-for-age, *SDS* standard deviation score based on published standards of the Dutch reference population [18]

### Polysomnography/ambulatory sleep study

Degree of OSA was determined using an overnight in-hospital polysomnography or an ambulatory sleep study. All sleep studies were scored according to the 2012 update of the American Association of Sleep Medicine (AASM) [18, 19]. Detailed scoring method used for both types of sleep studies have been described before [7, 12]. The obstructive apnea–hypopnea index (AHI) per hour was calculated by adding the number of obstructive apneas, mixed apneas, and obstructive hypopneas, divided by the total sleep time (TST). For OSA classification, the following criteria were used: mild = 1 ≤ AHI < 5; moderate = 5 ≤ AHI < 10; severe = AHI ≥ 10 [18].

### Statistical analysis

The descriptive statistics were reported as means as continuous variables and counts and proportions for categorical variables. In case of non-normally distributed data, the data was illustrated as medians with interquartile ranges (IQR). To assess the average growth parameters (WFA, WFH, and HFA), we calculated the weighted average per child. Using the weighted average, we account for the difference in the number of measurements per child, as children with a higher chance of malnutrition might undergo more weight and height measurements. To assess the effect of the degrees of OSA and OSA treatment on the growth parameters (WFA, WFH, and HFA), a univariate mixed-model was fitted with OSA and type of surgical treatment as time-varying covariates. To assess the effect of surgical treatment, a subset of all growth variables within 1 year before and 1 year after surgery were taken into account. For midface advancements, all growth variables within 1 year after removal of the distractors was used. Subject-specific random intercepts and random slopes were used to account for correlation between repeated measurements of the same child. Analysis was performed using the computing environment R 3.6.0 (R studio version 1.2.1335).

## Results

In total, 153 children with SCC were included in our study, of whom 617 height measurements and 958 weight measurements were retrieved. Baseline patient characteristics are provided in Table 2. The median follow-up time in our study population is 3.5 years (IQR; 0.87–5.56). In 37 children, the follow-up was less than 1 year, while 40 children had a follow-up > 5 years.

**Table 2:** Baseline patient characteristics (at intake)

	**Study population** (*n* = 153)
Age* (years) (median; IQR)	1.8 (0.50–5.1)
**Sex** (*n* patients) Male Female	84 80
**Syndrome** (*n* patients) Apert Crouzon Muenke Saethre-Chotzen Complex	24 38 23 32 47
**Treatment** A(T)E Midface advancement	39 25
**OSA**	
No OSA	109
Mild OSA	19
Moderate-severe OSA	25

*IQR* interquartile range, Age = at time of first measurement

**Fig. 1:**
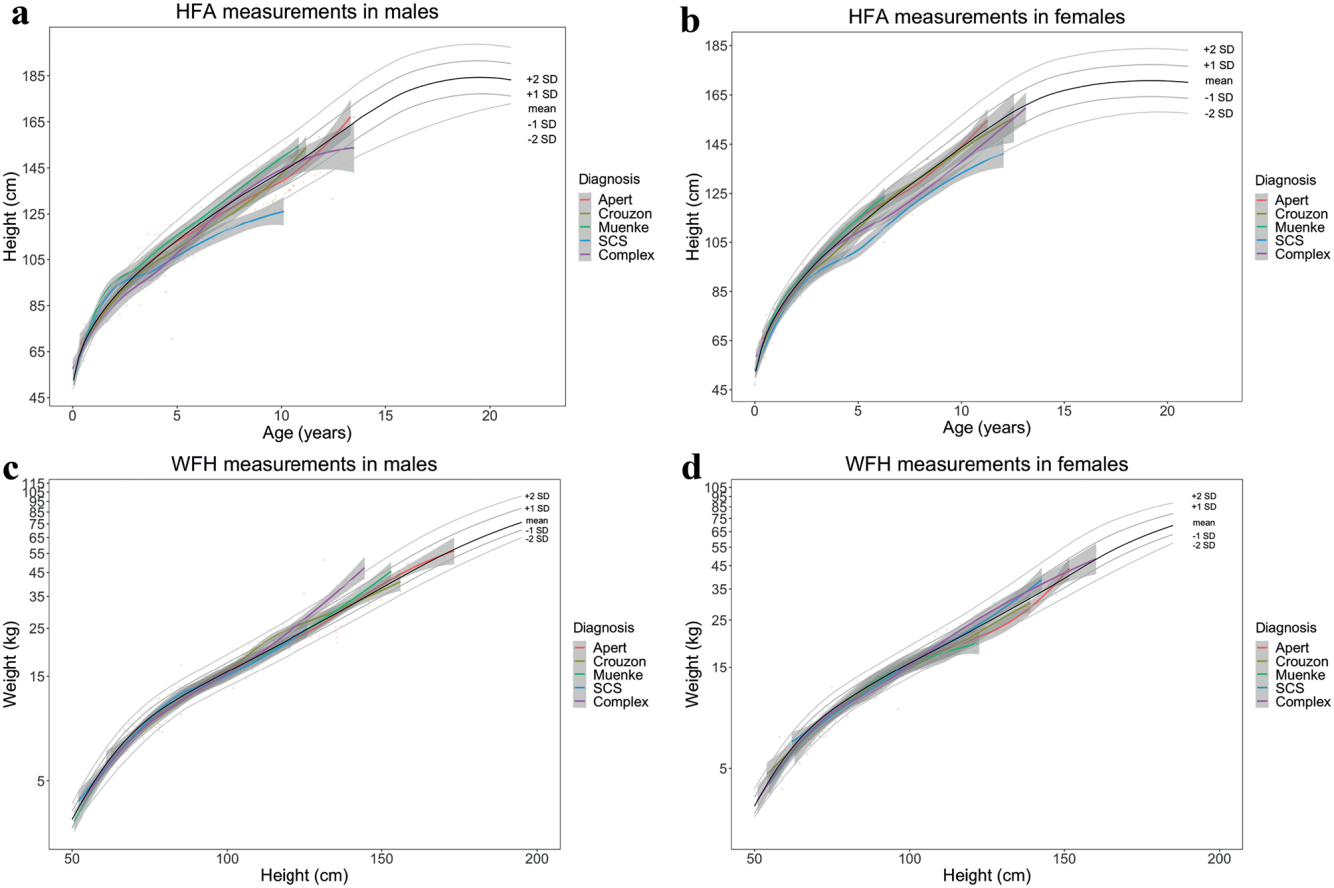
Growth chart of children with SCC without OSA, divided by syndrome and gender. Height-for-age SDS (**a**, **b**) and weight-for-height SDS (**c**, **d**) provided in males (**a**, **c**) and females (**b**, **d**) with SCC. The different syndromes are divided by color, with Apert (orange), Crouzon (yellow), Muenke (green), Saethre-Chotzen (blue), and complex (red)

**Fig. 2:**
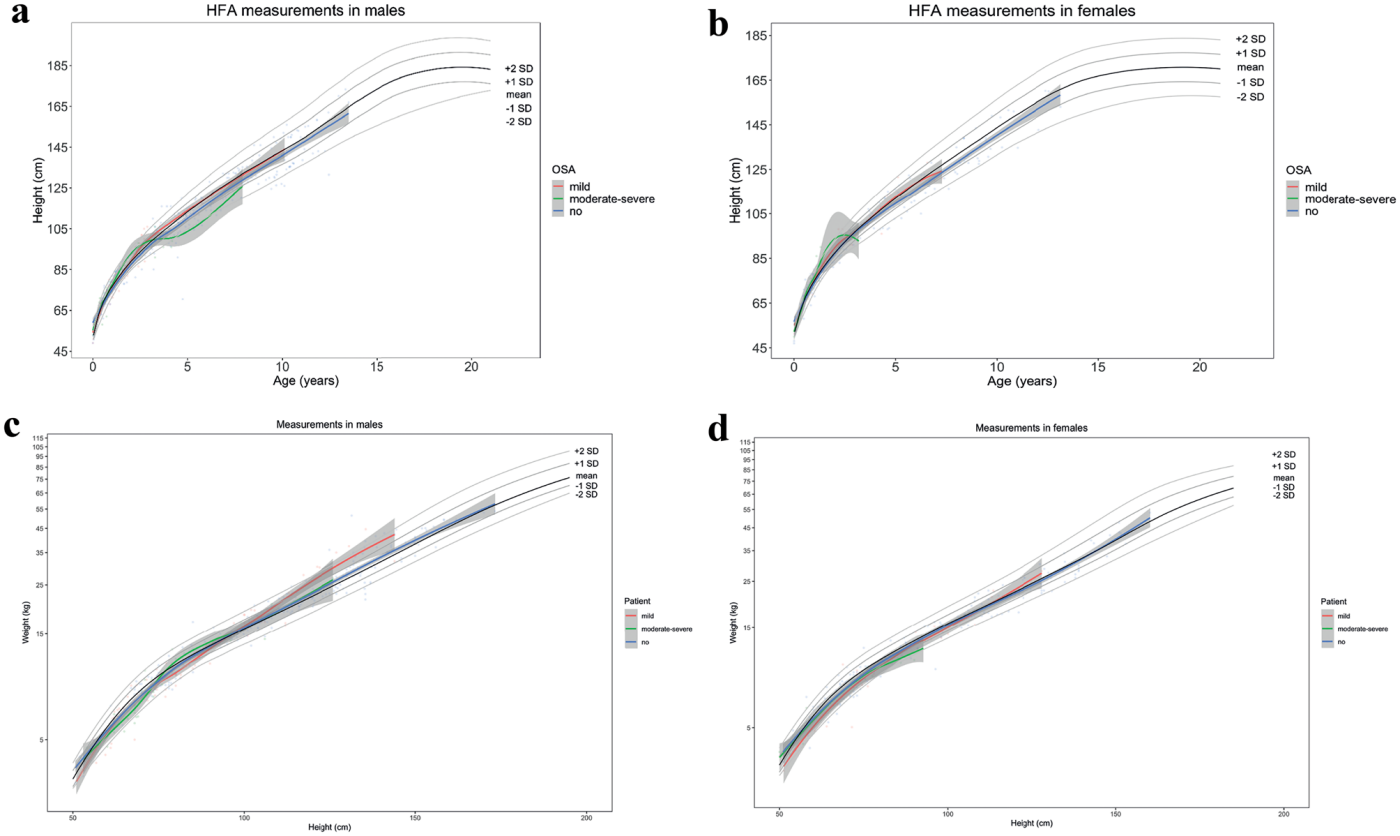
Growth chart of children with SCC, divided by in degree of OSA. Height-for-age SDS (**a**, **b**) and weight-for-height SDS (**c**, **d**) provided in males (**a**, **c**) and females (**b**, **d**) with SCC. The degrees of OSA are divided in no (blue), mild (red), and moderate-severe (green)

The weighted average of HFA SDS in children with SCC without OSA (*n* = 109) was −0.28 (IQR: −1.10 to 0.67), the weighted average of WFH SDS was −0.33 (IQR: −0.94 to 0.34), while the weighted average of WFA SDS in children < 1 year old was −0.33 (IQR: −1.00 to 0.48). Growth charts of children without OSA, are provided in Fig. 1, divided by type of syndrome. Growth charts of children divided by the degrees of OSA are provided in Fig. 2.

### Nutritional status and OSA

Overall malnutrition was seen at some point during follow-up in 67 of the 153 patients (43,8%); 38 patients had acute malnutrition (in 16 patients in combination with chronic) and 29 had solely chronic malnutrition. In 21 of the 38 patients, acute malnutrition was disease-related: in 2 patients after midface advancement surgery, in 1 patient after meningitis, in 1 patient after hospitalization due to craniosynostosis surgery, in 6 patients after upper respiratory tract infection, and in 11 patients who suffered from moderate-severe OSA.

Chronic malnutrition was based on the definition of < -2 HFA SDS in 23 children, of whom 12 also had a significant deflection of HFA SDS. At the end of follow-up, chronic malnutrition was still present in 7 children. Four of these 7 children had Saethre-Chotzen syndrome. The other three children had Apert syndrome with severe OSA, Crouzon syndrome with moderate OSA, and complex craniosynostosis with severe OSA. Of the 17 children who had both types of malnutrition, acute malnutrition was present prior to chronic malnutrition in 3 patients.

To assess the association between the degrees of OSA and the growth patterns (WFA, WFH, and HFA), a univariate mixed-model was fitted with OSA as time-varying covariate (Table 3). In our patient population, a significant difference in WFH SDS was seen between the children with moderate-severe OSA compared to children without OSA (*p* = 0.0063), regardless of age, diagnosis, and gender. Every year increase of age was significantly associated with a change in WFH SDS and HFA SDS (respectively, *p* = 0.0204, *p* = 0.0002), given that the degree of OSA, gender, and diagnosis were constant. Male gender was significantly associated with an increase of 0.47 in WFH SDS (*p* = 0.0200) compared to females. Children with Saethre-Chotzen syndrome (SCS) had significantly lower HFA SDS (*p* = 0.0096) compared to children with other types of SCC. Excluding children with SCS, the weighted average of HFA SDS in our patient population without OSA was 0.15 (IQR: −0.78 to 0.78).

**Table 3:** Linear mixed model with growth parameters

**Parameters**	**Growth parameters** (β, *p* value)			
	WFA SDS	WFH SDS	HFA SDS	HFA SDS*
OSA Mild Moderate-severe	−0.21 (0.43) 0.14 (0.57)	−0.06 (0.74) −0.55 **(*0.0063*)**	−0.16 (0.28) −0.09 (0.57)	−0.26 (0.44) 0.19 (0.23)
Diagnosis Apert Crouzon Muenke SCS	−0.29 (0.40) −0.37 (0.24) 0.08 (0.80) −0.40 (0.17)	0.46 (0.14) −0.14 (0.61) 0.01 (0.99) −0.30 (0.36)	−0.30 (0.33) −0.13 (0.66) 0.38 (0.29) −0.85 ***(0.0096)***	−0.24 (0.45) -0.06 (0.84) 0.31 (0.40)
Gender Male	−0.12 (0.56)	0.47 **(*0.0200*)**	−0.26 (0.22)	−0.41 (0.08)
Age	−0.17 (0.30)	0.07 (***0.0204)***	−0.07 ***(0.0002)***	−0.06 ***(0.0031)***

*WFA* weight-for-age, *WFH* weight-for-height, *HFA* height-for-age, *SDS* standard deviation score based on published standards of the Dutch reference population, age = age at time of measurement. The reference level for OSA was no OSA. The reference level for diagnosis was complex craniosynostosis. The reference level for gender was female. * Subset of the study population

### Effect of surgical treatment for OSA

Pre-operative and post-operative weight and height measurements were available in 47 children (ATE *n* = 29, midface advancement *n* = 18). The univariate mixed-model with surgical treatment as time-varying covariate is shown in Table 4. The median age at pre-operative weight measurement was 2.7 years (IQR: 1.11–3.35), while the median age at post-operative weight measurement was 3.43 years (IQR: 1.97–5.50). A significant increase in post-operative WFH SDS was present after ATE, compared to the pre-operative WFH SDS (*p* = 0.044). This change in WFH SDS was not seen after midface advancement.

**Table 4:** Effect of surgical treatment on growth

**Parameters**	**WFH SDS**	
	β	*p* value
Treatment		
ATE	0.313	***0.04***
Midface advancement	-0.115	0.54
Gender		
Male	0.63	0.05
Age	0.05	0.51

## Discussion

In this study, we found that from the 153 studied children with SCC, 38 (25%) were acutely malnourished at some point during follow-up. Twenty-one of them had disease-related acute malnutrition. Children with moderate-severe OSA had significant lower weight-for-height SDS compared to children without OSA, regardless of age, gender, and type of syndrome. Furthermore, the growth parameters weight-for-age SDS, weight-for-height SDS, and height-for-age SDS in children with SCC without OSA was not impaired as it did not differ from the healthy population. Exception were patients with Saethre-Chotzen syndrome (SCS) who had a lower height-for-age (HFA) SDS, which can be attributed due to their genetic mutation (*TWIST1*) [20].

Overall, children with SCC with a prolonged period of moderate-severe OSA had a significantly lower weight-for-height (WFH) SDS compared to children without OSA. This result is consistent with the literature in other groups of children with OSA, which illustrates that the presence of OSA is associated with weight-loss and faltering growth. A higher energy expenditure in these patients is an important causative factor [21–23]. Additionally, the significant increase in post-operative WFH SDS after ATE noticed in our patient population is also in accordance with the literature, as weight gain and catch-up growth are reported after resolution of OSA [24, 25]. These results indicate the importance of assessing weight and growth patterns in patients who are clinically suspected for moderate-severe OSA.

Concerning chronic malnutrition, impaired growth in length can be caused by a prolonged period of acute malnutrition and additionally by an impaired secretion of growth hormones (IGF-1) which can be present in patients with OSA [26]. In our study, chronic malnutrition was seen in 45 children at some time point during follow-up. In only 3 children, a period of acute malnutrition was present prior to chronic malnutrition. Although children with moderate-severe OSA had a lower weight-for-height SD score, no significant association between the presence of OSA and change in height-for-age was found. A probable explanation could be due to the fact that our OSA screening and treatment protocol by a multidisciplinary team has been appropriate enough to prevent long-term impaired growth in height [12, 27]. However, at the end of follow-up, chronic malnutrition was still present in 7 children. Four children had Saethre-Chotzen syndrome, which could explain their significant lower height. In the other 3 children, no explanation for their lower height was found. Further research is needed in terms of the potential causes of chronic malnutrition in children with SCC.

In contrast to the significant difference in WFH SDS found in children with moderate-severe OSA, no significant difference was seen in patients with no or mild OSA compared to the healthy population. Treatment of mild OSA has been a subject of discussion in the general pediatric population, as spontaneous resolution of mild OSA was seen in 65% of the subjects in the CHAT study [28]. This spontaneous resolution of mild OSA was also seen in children with SCC [1, 12].

This study had two limitations. First, a number of growth measurements were performed in between polysomnographies, as the number of weight and height measurements were higher than the number of performed polysomnographies. Hence, the degree of OSA of these aforementioned growth measurements were assumed based on the previous polysomnographies. Theoretically this might lead to an underestimation of the effect of OSA on growth. Secondly, a limitation would be the small number of children with mild or moderate-severe OSA which might have influenced the results. However, to our knowledge, this is the first study about growth patterns and the effect of OSA on growth in children with SCC.

In conclusion, children with SCC have a substantial chance of developing acute malnutrition at some point during growth. Additionally, in children with moderate-severe OSA, a significant lower SDS for weight-for-height is present, indicating the importance of assessing the weight and growth pattern in patients who are clinically suspected for OSA. With regard to length growth, no significant difference in height-for-age SDS is seen in children with moderate-severe OSA, indicating that the treatment in our patient population with OSA is reliable and adequate.

## Abbreviations

AHI: Obstructive apnea–hypopnea index
ATE: Adenotonsillectomy
HFA: Height-for-age
HSAD: Home sleep apnea device
IQR: Interquartile range
OSA: Obstructive sleep apnea
PSG: Polysomnography
SCC: Syndromic and/or complex craniosynostosis
SCS: Saethre-Chotzen syndrome
SDS: Standard deviation scores
TST: Total sleep time
WFA: Weight-for-age
WFH: Weight-for-height
